# Chemical Profile, Antibacterial and Antioxidant Activity of Algerian Citrus Essential Oils and Their Application in *Sardina pilchardus*

**DOI:** 10.3390/foods4020208

**Published:** 2015-06-05

**Authors:** Djamel Djenane

**Affiliations:** Laboratory of Food Quality and Food Safety, Department of Food Science, University Mouloud MAMMERI, BP 17, Tizi-Ouzou 15000, Algeria; E-Mail: d.djenane@hotmail.com; Tel.: +213-779-001-384; Fax: +213-26-18-61-56

**Keywords:** citrus, essential oils, GC-MS, antibacterial and antioxidant activity, *Staphylococcus aureus*, *Sardina pilchardus*

## Abstract

Stored fish are frequently contaminated by foodborne pathogens. Lipid oxidation and microbial growth during storage are also important factors in the shelf-life of fresh fish. In order to ensure the safety of fish items, there is a need for control measures which are effective through natural inhibitory antimicrobials. It is also necessary to determine the efficacy of these products for fish protection against oxidative damage, to avoid deleterious changes and loss of commercial and nutritional value. Some synthetic chemicals used as preservatives have been reported to cause harmful effects to the environment and the consumers. The present investigation reports on the extraction by hydrodistillation and the chemical composition of three citrus peel essential oils (EOs): orange (*Citrus sinensis* L.), lemon (*Citrus limonum* L.) and bergamot (*Citrus aurantium* L.) from Algeria. Yields for EOs were between 0.50% and 0.70%. The chemical composition of these EOs was determined by gas chromatography coupled with mass spectrometry (GC/MS). The results showed that the studied oils are made up mainly of limonene (77.37%) for orange essential oil (EO); linalyl acetate (37.28%), linalool (23.36%), for bergamot EO; and finally limonene (51.39%), β-pinene (17.04%) and γ-terpinene (13.46%) for lemon EO. The *in vitro* antimicrobial activity of the EOs was evaluated against *Staphylococcus aureus* (*S. aureus*) using the agar diffusion technique. Results revealed that lemon EO had more antibacterial effects than that from other EOs. Minimal inhibitory concentrations (MICs) showed a range of 0.25–0.40 μL/mL. Lemon and bergamot citrus peel EOs were added at 1 × MIC and 4 × MIC values to *Sardina pilchardus* (*S. pilchardus*) experimentally inoculated with *S. aureus* at a level of 3.5 log_10_ CFU/g and stored at 8 ± 1 °C. The results obtained revealed that the 4 × MIC value of bergamot reduced completely the growth of *S. aureus* from day 2 until the end of storage. The presence of EOs significantly extended lipid stability. Samples treated with bergamot EO displayed greater antioxidant activity than lemon EO. In fact, the oxidation rate is inversely proportional to the concentration of EO. At 1 × MIC and 4 × MIC values of bergamot EO, the levels of malonaldehyde compared to the control samples were 1.66 and 1.28 mg malonaldehyde/kg at the end of storage, corresponding to inhibition percentages of 42.76% and 55.87%, respectively. These results suggest the possibility that citrus EOs could be used as a way of combating the growth of common causes of food poisoning and used as potent natural preservatives to contribute to the reduction of lipid oxidation in sardines.

## 1. Introduction

The nutritional importance of fish is associated with its omega-3 (ω-3) fatty acid content. Increasing the dietary intake of eicosapentaenoic acid (EPA) and docosahexaenoic acid (DHA) has a positive effect on several diseases. The variation of fatty acid composition of sardines was determined in relation to season and site of catch in the Algerian Mediterranean Sea [1]. However, fish fats are the most sensitive of the nutritional fats to oxidation during storage [2,3,4].

Food safety is a fundamental concern of both consumers and the food industry, especially as the number of reported cases of food-associated infections continues to increase. The presence of *S. aureus* in foods is related to improper handling by personnel and fresh meat contaminations from animal origins are becoming more frequent. The typical scenario for staphylococcal food poisoning is contamination of a heat-treated food, abolishing most of the competing bacteria and, together with cooling failure, providing ideal conditions for growth of staphylococci.

Recently, consumers health concerns in relation to food ingredients have led to an increase in the request of foods processed without the addition of synthetic chemical preservatives. The use of EOs may provide a “natural” alternative to the chemical preservation of foods. Novel or emerging non-thermal food processing technologies including natural antimicrobials are gaining increasing importance [5,6].

Citrus EOs have been industrially applied in many products, including foods and beverages [7,8,9] and the activities against some of the most important foodborne pathogens have been proven [10,11,12,13]. Since citrus EOs are mainly located in the fruit peel, their extraction is economically sustainable, because the fruit peel constitutes a waste for the fruit juice industry [14].

In the Algerian economy, citrus EOs have great potential to meet the demands of the food, pharmaceutical and cosmetic industries, since they are not only easy and inexpensive to produce but are also without any perceivable hazard for humans. EO extracted from citrus by-product can be used in food as flavoring ingredients and pharmaceutical industries for its anti-inflammatory and antibacterial effects. In addition, substantial quantity of this oil is also used in the preparation of toilet soaps, perfumes and cosmetics.

The objectives of this study were to evaluate the chemical composition of the EOs extracted from fruit peel of several citrus varieties and to determine their antibacterial activity against *S. aureus* as well as to analyze antioxidant activity of the most “*in vitro*” effective EOs in sardine.

## 2. Results and Discussion

### 2.1. Yields and Chemical Constituents of Essential Oils

The results showed that the average yield (volume of the oil/total weight of peel) in EOs of bergamot, lemon and orange are in the order of 0.60%, 0.70% and 0.58%, respectively. The results obtained in this work (Table 1) are similar to the reuslts of others. Indeed, Jeannot *et al*. [15] reported yields ranging from 0.25% to 0.57% for bergamot EO. However, Fisher *et al*. [16] and Eleni *et al*. [17] reported that yields in citrus EOs differ depending on the species and reported yields of 1% to 3%. This difference could be explained according to Kelen and Tepe [18] by the choice of the harvest period because it is critical in terms of yield and quality of EO. Sarrou *et al*. [19] reported yields in bergamot EO of 0.12%, 1.67%, 0.27% and 0.45%, depending on the source hydrodistilled flowers, peel, and young and old leaves. Climate, geography, genetics of the plant, the organ of the plant used, the degree of freshness, the drying period, the extraction method used, *etc.* are considered among other factors that may have a direct impact on the yields of EOs obtained. To explain the impact of the extraction technique used on EO yield, ultimately, our group found zero yields of *Myrtus communis* and *Eucalyptus globulus* EOs by steam distillation at laboratory scale; however, the EO yields of these two plants were improved to 0.05% and 0.06%, respectively, when the extraction was carried out using a semi-industrial hydrodistillator (no published data).

**Table 1:** Chemical Composition (%) of bergamot, lemon and orange EOs acclimated to Algeria from fresh peel parts as identified by GC-MS analysis.

No.	Compounds ^*,•,▪^	RI ^a^	Percentage (%) ^b^		
			Bergamot EO	Lemon EO	Orange EO
**1**	α-Pinene	935	00.77	03.07	00.22
**2**	β-Phellandrene	964	01.92	-	-
**3**	**β-Pinene**	980	03.45	**17.04**	01.62
**4**	β-Myrcene	990	-	02.37	03.20
**5**	Octanal	1006	01.25	-	-
**6**	Carene	1020	-	-	01.09
**7**	**Limonene**	1033	**77.37**	**51.40**	02.20
**8**	Ocimene	1048	-	-	02.24
**9**	**γ-Terpinene**	1060	-	**13.46**	-
**10**	**β-Linalool**	1080	01.22	-	**23.37**
**11**	Decanal	1175	00.83	-	-
**12**	Nerol	1207	-	01.50	-
**13**	Geraniol	1234	-	02.43	-
**14**	**Linanyle acetate**	1255	-	-	**37.29**
**15**	Citral	1336	-	-	00.34
**16**	Neryl acetate	1365	-	01.05	04.10
**17**	Geranyl acetate	1384	-	-	06.35
**18**	Caryophyllene	1428	-	-	01.12
**19**	Naphtalene	-	01.42	-	-
**20**	Isocaryophyllene	-	-	01.23	-
Total identified components (%)			**88.21**	**93.55**	**83.14**
Yields (%)			**0.60**	**0.70**	**0.58**

^a^ RI: Retention index relative to *n*-alkanes (C7-C29) on non-polar HP5MS capillary column; ^b^ Compounds present in trace amounts (<0.10%) were not registered; ^*^ Identification by Kovats index (Adams, 2001) [64]; ^•^ Identification by NIST MS library; ^▪^ Identification by authentic standards analyzed by mass spectrometry.

**Figure 1:**
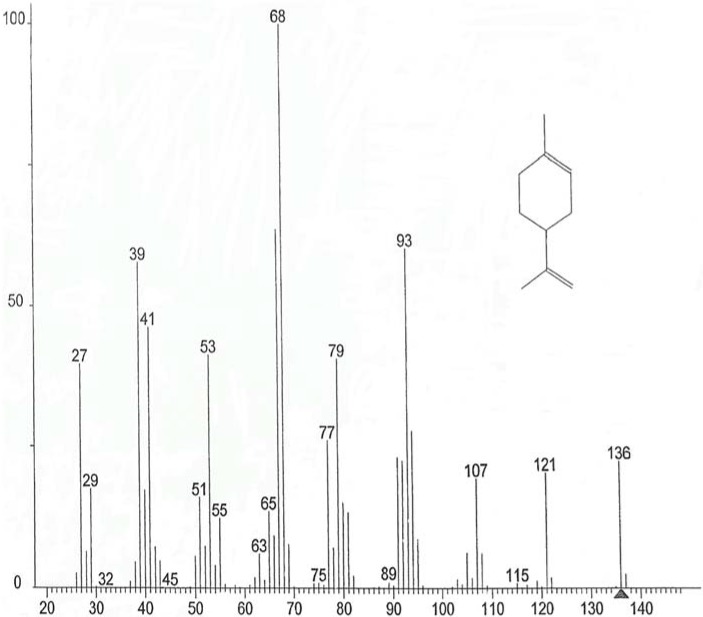
Mass spectra of limonene.

**Figure 2:**
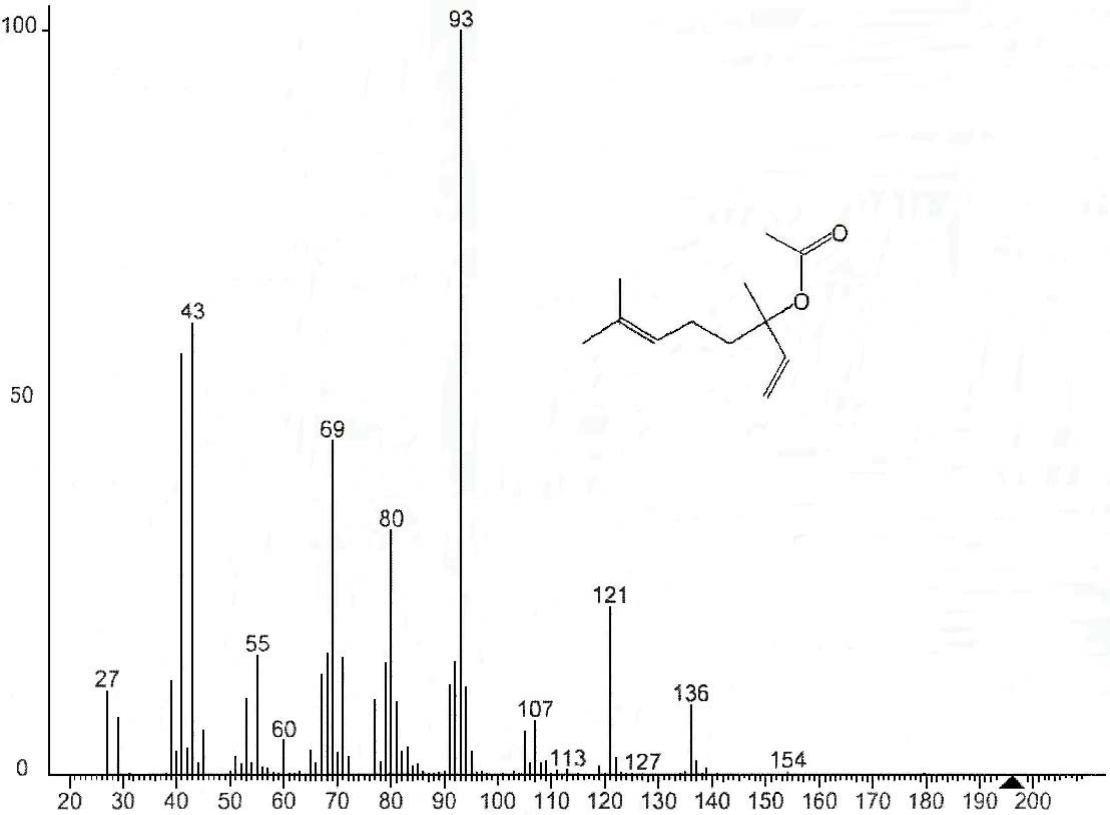
Mass spectra of linalyl acetate.

**Figure 3:**
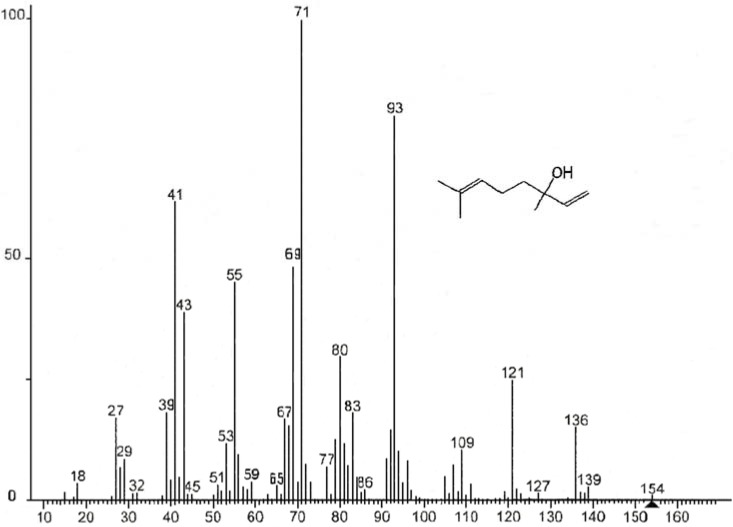
Mass spectra of linalool.

**Figure 4:**
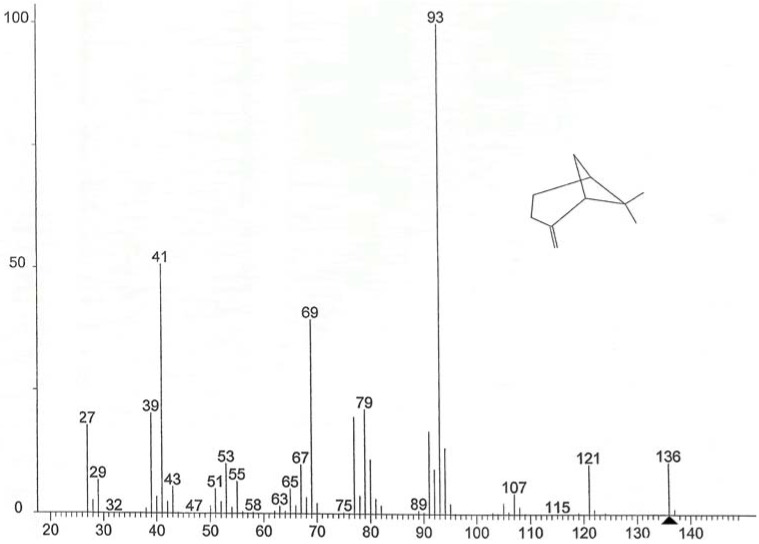
Mass spectra of β-pinene.

The components of EOs are important, as their qualitative and quantitative composition determines the characteristics of the oils and subsequent effect on its antimicrobial potential. Chemical analysis showed the number of components determined for the three EOs (Table 1), which represented 88.21%, 93.55% and 83.14% of the total EO of bergamot, lemon and orange, respectively. This analysis shows that on the one hand, most of the identified substances are monoterpene hydrocarbons, and secondly limonene (mass spectra see Figure 1) is undoubtedly the major component EO of orange (77.37%) and lemon (51.40%). Conversely EO of bergamot is dominated by the presence of two major compounds: linanyle acetate (mass spectra see Figure 2) (37.30%) and linalool (mass spectra see Figure 3) (23.37%). These two components alone constitute 60.65% of the total identified. Other monoterpene compounds identified in the same EO have significant levels as geranyl acetate (6.35%), neryl acetate (4.10%), β-myrcene (3.20%), ocimene (2.24%), and in smaller quantities include β-pinene (1.62%), caryophyllene (1.11%), carene (1.08%), α-pinene (0.22%) and citral (0.34%). In addition limonene is the major component of orange oil, other compounds such as β-pinene (mass spectral see Figure 4) (3.45%), β-phellandrene (1.92%), naphthalene (1.42%), octanal (1.24%), linalool (1.21%), and some form of traces decanal (0.83%) and α-pinene (0.77%) are also present in this oil. Similar findings have been reported by Eleni *et al*. [17]; and Aazza *et al*. [20] who reported that bergamot EOs consist mainly of linalyl acetate and linalool.

Our results are quite different from those obtained by Svoboda and Greenaway [21] on the orange (Osbeck Hongiian variety from China). The chemical composition of this EO consists mainly of limonene (93.60%) and β-myrcene (2.00%), and minor compounds such as decanal (0.82%), sabinene (0.70%), α-pinene (0.40%) and β-phellandrene (0.30%), totaling 88.21%. By studying the chemical composition of EOs of orange and lemon, Moufida and Marzouk [22] confirmed that these EOs consist mainly of limonene. This compound varies between 68%–98% for orange and 45%–76% for bergamot, and linalool that is shown at low levels 0.20% and 10.23% respectively, and compounds of neral/geranial (together are often referred to citral) are present in lemon, orange and bergamot EOs at concentrations ranging from 0.10%, 0.70% and 3.00% respectively.

Chemical analysis of lemon EO has identified as the major compounds limonene, β-pinene and γ-terpinene with percentages of 51.40%, 17.04%, and 13.46%, respectively, followed by other molecules in low concentrations: α-pinene (3.07%), geraniol (2.43%), β-myrcene (2.37%), nerol (1.50%), isocaryophyllene (1.23%) and neryl acetate (1.05%). Our results on the chemical profile of the oil of lemon agreed with those of Vekiari *et al*. [23] who showed that the lemon EOs are characterized by high concentrations of β-pinene, γ-terpinene and α-pinene (21.20%, 17.40% and 9.80%) respectively. It should be noted from this analysis that the acyclic compounds such as nerol and geraniol are identified only in the lemon EO. In agreement with our results, Gancel *et al*. [24] noted the absence of these two compounds in the species orange and bergamot. Several investigations on the determination of the chemical composition of citrus EOs were performed by Moufida and Marzouk [22]; Belletti *et al*. [25]; and Rehman *et al*. [26] and the results have shown that these EOs usually consist mainly of monoterpene compounds (97%), whereas other compounds, such as alcohols, aldehydes and esters, are represented with low contents from 1.80% to 2.20%.

By studying the chemical composition of citrus EOs, Sharma and Tripathi [27] concluded that in addition to monoterpenes, these EOs contain fatty acids in relatively low amounts (0.8%). Linolenic acid is the main fatty acid of orange; linoleic acid is found in lemon and oleic acid in bergamot. Flavonoids are found in citrus oils and non-volatile component of the oils and are useful in the differentiation between species of citrus. It was established that the chemical composition and the content of different components between geographical regions and even between different specimens of a species of the same region. Several research studies have been studying different chemotypes of citrus EOs [28,29,30,31,32,33]. In fact, these authors reported that lemon EO might be chemotypes β-pinene, limonene, linalool, linalyl acetate, citral or citronellal; bergamot EO might be chemotypes limonene, linalool or decanal and orange EO might be chemotypes D-limonene, geraniol, linalool, citral, citronellal, terpineol or decanal.

To explain this differentiation between the chemical profile EOs, Senatore *et al*. [34] indicated that the qualitative and quantitative variations found in the chemical composition of EOs, may depend on one or a combination of three factors: genetic, age and the environment of the plant.

It should also be noted that the maturity of the fruit has a significant impact on the chemical composition of EO obtained. Thus, terpenes are exclusively present in the EO of immature fruit, and as the fruit matures, concentrations of aldehydes, terpenes and aliphatic oxygenated sesquiterpenes increase [35].

### 2.2. Screening of Essential Oils (Disc Assay)

**Figure 5:**
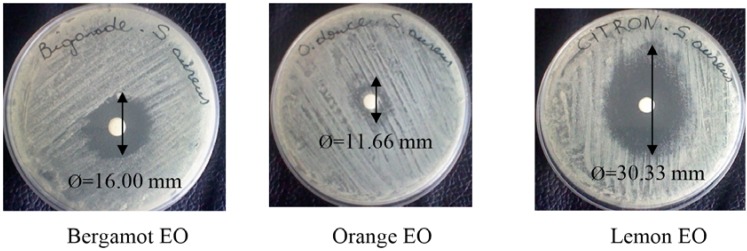
Antibacterial activity (*S. aureus*) of the EOs from citrus, using paper disc-diffusion method, expressed by diameter (mm) of inhibition zone (including the disc diameter, 6 mm).

The EO of orange showed the lowest antibacterial activity compared to bergamot and lemon EOs, with average diameter inhibition of 11.66 mm (Table 2). EO of bergamot presented moderate antibacterial activity, with the diameter of the inhibition zone at 16 mm. It is noteworthy that the highest antimicrobial activity was recorded with lemon EO with an average value of 30.33 mm (Figure 5). These results are consistent with the work of Djenane *et al.* [36,37] who tested the antibacterial effect of extracts of plants against several Gram positive and Gram negative. The results showed that *S. aureus* was more sensitive than *Salmonella* Enteritidis and *Esherichia coli*. Billerbeck [38] reported that *S. aureus* presented a variable sensitivity to some citrus EOs species. Similar results were recorded with other types of EOs by Akin *et al*. [39]. Testing the EO of *Eucalyptus camaldulensis* against *S. aureus*, *P. aeruginosa* and *E. coli*, the same authors reported that *S. aureus* was the only one to show some sensitivity to EO. Smith-Palmer *et al*. [40], using the method of well on agar medium, have reported that lime EO was more effective against *S*. Enteritidis (Gram negative) than lemon EO on *S. aureus*.

**Table 2:** Antibacterial activity of the EOs from citrus, using a paper disc-diffusion method, expressed by diameter (mm) of inhibition zone (including the disc diameter, 6 mm).

	Ø ^a^ (mm)			
	Lemon EO	Bergamot EO	Orange EO	Chloramphenicol
*S. aureus* CECT 4459	30.33 ± 1.53 ^x^	16.00 ± 1.00 ^y^	11.66±1.50 ^z^	21.42 ± 0.90 ^w^

Values followed by the different letter (x, y, z and w) under the same line are significantly different (*p* < 0.05). ^a^ ϕ: Inhibition zone in diameter around the discs impregnated with EOs. The diameter (6 mm) of the disc is included. All tests were performed in triplicate.

Delaquis *et al*. [41] and Burt [42] tried to explain this differential sensitivity between bacteria (Gram positive and Gram negative). It is noteworthy that no significant correlation was found between the content of chemical components and antibacterial activity of citrus EOs. Indeed, limonene is the major component of orange EO. It appears that orange EO, which contained 77.40% limonene, was less effective as an antimicrobial than bregamot EO, which contained only 2.20% limonene. Based on these observations, it is clear that limonene had no influence on the antibacterial potential of the EOs. These observations concerning the correlation antimicrobial activity with the major compounds confirmed our previous study [43]. The compounds present in larger proportions may not necessarily be responsible for the antibacterial activity of EOs. Thus the participation of less abundant constituents must be considered [41]. According to the investigations of different authors [44,45], the antimicrobial activity of some EOs could be attributed to the presence of minor compounds known to exhibit antibacterial activity involved in the phenomena of synergy between the various components which may cause a much more pronounced antimicrobial effects than that expected by the major compounds. The antibacterial properties of these compounds are partly related to their lipophilicity leading to accumulation in bacterial walls, thereby interfering with the operation and the permeability of cell membranes.

In order to improve d-limonene antimicrobial activity, a number of approaches have been explored. It was reported that combinations of nanoemulsion d-limonene [46,47] or with other natural antibacterial compounds (e.g., nisin) could achieve effective antimicrobial activity at sufficiently low dosages and remarkably reduce the negative sensory impact on foods [46].

It should be noted that there was considerable variation between the antimicrobial activities of several species of plants. However, a comparison of the effectiveness of EOs through different publications is difficult. The chemical composition of EOs varies depending on the environmental conditions of the plant, even within the same species. This means the antimicrobial properties of EOs may change as their chemical composition and the relative proportions of the various constituents are influenced by genotypes, chemotypes, and the method used to assess the antimicrobial activity (the technique of agar disk diffusion or by dilution method); the results obtained by each of these methods may be different, depending on the choice of organism, microorganism growth, the period of exposure of the microorganism to the EO, and the choice of the emulsifier to solubilize the EOs. These are all factors that may explain conflicting results from different studies.

### 2.3. Microdilution Assay

The results of MICs of EOs tested are shown in Table 3. The results of the agar diffusion showed that among the three EOs tested, the EO of orange is characterized by low antimicrobial activity compared to other oils. However the EOs lemon and bergamot revealed an interesting antibacterial activity. MICs were calculated only for EOs which have previously exhibited a significant antibacterial effect. Thus, lemon and bergamot EOs were selected for study because of their reported antimicrobial activity.

**Table 3:** Minimal inhibitory concentrations values from citrus EOs using broth microdilution method.

	MIC (μL/mL) *		
	Lemon EO	Bergamot EO	Chloramphenicol
*S. aureus* CECT 4459	0.25	0.40	0.15

* Minimal inhibitory concentration values expressed by μL/mL. All tests were performed in triplicate.

The bergamot EO showed an inhibitory effect on the dilution of 0.40 μL/mL (0.04%), and the inhibitory effect of lemon EO was observed until the dilution corresponded to 0.03% (0.25 μL/mL). According to the MIC values obtained, lemon EO showed better antimicrobial performance compared to the bergamot EO against *S. aureus*. Oussalah *et al*. [48] reported that among the twenty eight (28) EOs tested, twenty-six (26) exhibited an MIC ≤ 0.40% against the target bacterium. Smith-Palmer *et al*. [40] studied the antimicrobial activity of 2 citrus EOs (sweet orange and lime) against two types of bacteria, Gram negative (*S*. Enteritidis) and Gram-positive (*S. aureus*), using the agar diffusion method in wells. The results obtained showed that sweet orange EO is active against *S*. Enteritidis, and the lime has an antibacterial effect against *S. aureus* with MICs of 6.40 mg/mL and 12.80 mg/mL respectively. For its part, the antibacterial effect of the EO of lemon was also tested against *E. coli* by Moreira *et al*. [49]. The results showed MIC values of 2.50 mg/mL. Nezar *et al*. [50] evaluated the most active components of citrus EOs, in this case citral (0.50%), and linalool (3.50%) against *Shigella sonnei* and *Shigella flexneri*. Citral reduced approximately 10% of bacterial load, while linalool was completely inhibited both strains after only two hours of direct contact. Also, the antibacterial effect of lemon, orange, bergamot EOs and their components; linalool and citral were studied by direct contact *in vitro* against *Campylobacter jejuni*, *Esherichia coli* O157 H: 7, *Listeria monocytogenes*. Linalool and citral presented MICs of 0.13%, 0.06% and 0.05% (*v*/*v*), respectively [8,51].

### 2.4. Application in Sardine

#### 2.4.1. Antimicrobial Activity

**Figure 6:**
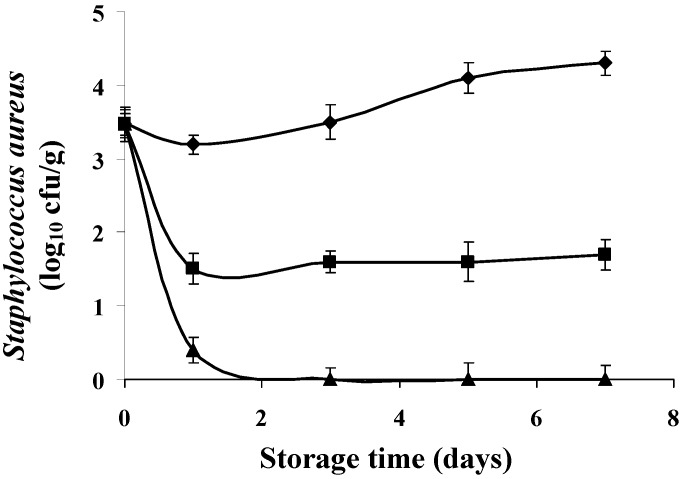
Inhibition growth of *S. aureus* by bergamot EO at different concentrations in *Sardina pilchardus* stored at 8 ± 1 °C under aerobic conditions. (◆) Control; (■) *C. aurantium* (1 × MIC value); (▲) *C. aurantium* (4 × MIC value). Error bar represents the standard deviation for triplicate experiments.

**Figure 7:**
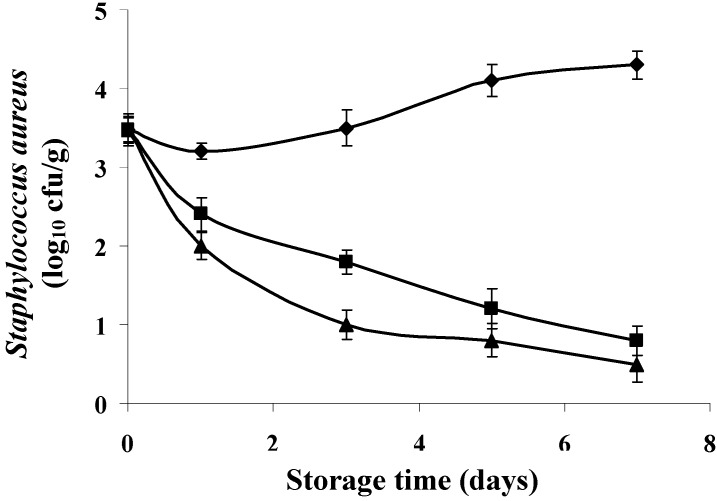
Inhibition growth of *S. aureus* by lemon EO at different concentrations in *Sardina pilchardus* stored at 8 ± 1 °C under aerobic conditions. (◆) Control; (■) *C. limonum* (1 × MIC value); (▲) *C. limonum* (4 × MIC value). Error bar represents the standard deviation for triplicate experiments.

Figure 6 and Figure 7 shows the results of development of *S. aureus* on fresh sardines stored at 8 ± 1 °C in the presence of different concentrations of bergamot and lemon EOs. According to the results, both EOs were effective against *S. aureus* (*p* < 0.05). However, the effect of the concentration is crucial. Application of lemon EO at 4 × MIC values resulted in a considerable reduction in growth of *S. aureus*. Indeed, a reduction of 2.50 log_10_ cfu/g was recorded during the third day of storage. However, the reductions achieved are 3.30 (80.48% reduction), 3.80 (88.37% reduction) log_10_ cfu/g during the fifth and seventh day of storage compared to controls.

It is evident from our results that application of bergamot EO (4 × MIC) has a more pronounced effect on bacterial reduction compared to the lemon EO. Figure 6 shows that from the 2nd day and throughout the storage period, the number of *S. aureus* reached zero values (bactericidal activity).

 It should be noted that the “*in vitro*” antibacterial effect of two EOs and upon application in the food matrix has different effect but both reduce the level of contamination at both concentrations tested compared to the controls.

The EO of bergamot seems more appropriate for application to the sardine. In recent years, some studies have successfully demonstrated the potential applications of EOs to reduce or control the presence of pathogens in food products, such as fish [8,37,43,52]. Indeed, according to Tassou *et al.* [53], phenolic extracts were applied at a concentration of 0.4% to a tuna salad inoculated with 7 log_10_ cfu/g of the different microorganisms. The results have shown that these extracts have variable antimicrobial activity according to the target bacterium. The authors reported a reduction of 0.50, 0.90 and 2 log10 cfu/g, respectively, for *S. aureus*, *Pseudomonas fragi* and *S*. Enteritidis. Mahmoud *et al*. [54] studied the antibacterial activity of the two components in abundant lemon and orange (citral and linalool) at a concentration of 2% at 2 °C for 48 h on the surface of the fish microflora. Linalool is more effective against *Acinetobacter* spp.; *Enterobacteriaceae*, *Morexella* spp. and *Vibrionaceae*, whereas citral has allowed a reduction of 1.50 log_10_ cfu/g of *S*. Typhimurium.

It is believed that the antimicrobial activity of EOs is mainly due to the presence of several compounds. Lin *et al*. [55] showed that several chemical compounds found in the EOs have an antibacterial effect. For their part, Oussalah *et al*. [56] reported that 1,8-cineol, α-pinene, limonene, and α-thujone are among the most active antimicrobial compounds found in various EOs. An important characteristic of its terpenoid molecules is their hydrophobicity, which allows them to partition into lipid membranes of bacterial cells and mitochondria, structures affecting bacterial membranes and making them more permeable [42]. According to Inouye *et al*. [57], the inhibitory activity of the volatile components such as linalool, citral, and limonene occurs in the first 3 h and then rapidly decreases over time. It was established that the antimicrobial effect of EOs is conditioned by several factors such as the availability of nutrients in foods that can quickly repair damaged cells; the presence of fat/protein/antibacterial substances, salt and other substances; and the pH, temperature, type of packaging, and the characteristics of the microorganism. Mejlholm and Dalgaard [52] have shown that low pH and the presence of salt increases the hydrophobicity of EOs, which facilitates their dissolution in the phospholipid of the membranes. Meanwhile, proteins and fats in foods protect the bacteria against the action of EOs; carbohydrates appear to have little or no influence on this activity.

#### 2.4.2. Antioxidant Activity

**Figure 8:**
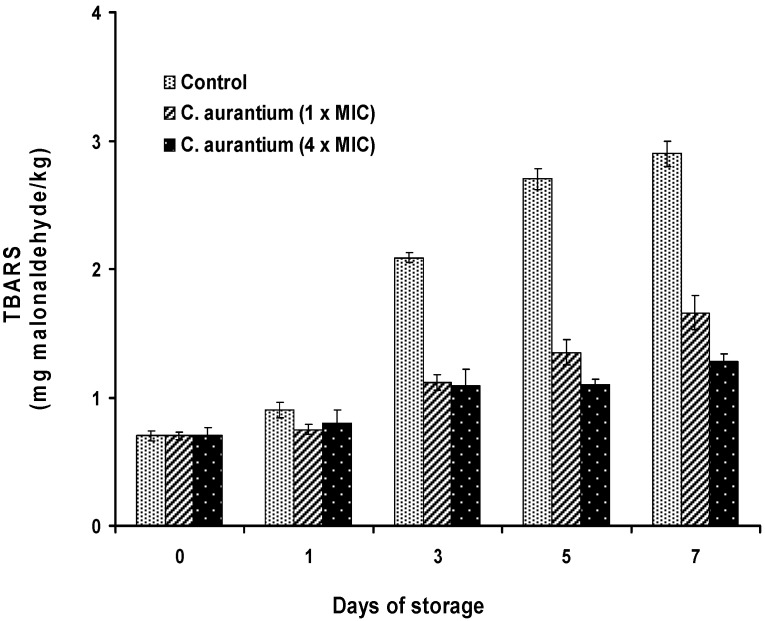
TBA-RS (mg malondialdehyde/kg) in *Sardina pilchardus* treated with bergamot EO at different concentrations and stored at 8 ± 1 °C under aerobic conditions. Error bar represents the standard deviation for triplicate experiments.

**Figure 9:**
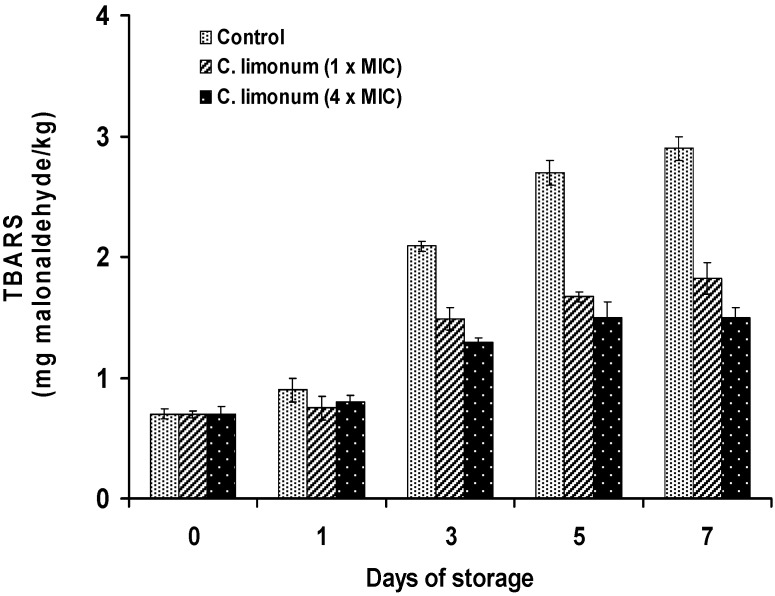
TBA-RS (mg malondialdehyde/kg) in *Sardina pilchardus* treated with lemon EO at different concentrations and stored at 8 ± 1 °C under aerobic conditions. Error bar represents the standard deviation for triplicate experiments.

The results obtained by the TBA-RS test (Figure 8 and Figure 9) showed that the antioxidant activity of lemon and bergamot EOs is higher in treated samples compared to the control (*p* < 0.05). The treatment with the oils resulted in a reduction of lipid oxidation after the first day of storage. Samples treated with bergamot EO displayed greater antioxidant activity than lemon EO. In fact, the oxidation rate is inversely proportional to the concentration of EO. At 1 × MIC and 4 × MIC values of bergamot EO, the levels of malonaldehyde compared to the control samples were 1.66 and 1.28 mg malonaldehyde/kg at the end of storage, corresponding to inhibition percentages of 42.76% and 55.87%, respectively. The sardine is a product highly exposed to oxidation processes. However, the addition of citrus oils can provide sufficient protection. The different activities observed may be due to the difference in the chemical composition of EOs. Although the antioxidant activity of bergamot EO may be related to the content of the main component, it should not be overlooked as the effect of other minor components. Lemon does not contain the same components as bergamot, which may explain its performance as a less effective antioxidant.

The inhibitory activity of herbal extracts is basically attributed to their richness in phenolic compounds [5,58]. Various extracts of herbs and EOs are potential sources of natural chemicals responsible for antioxidant activity. Concerning this subject, citrus fruit EOs and their constituents are recognized as GRAS [59] and their flavors lend themselves to use in food [60]. For this purpose several tests were conducted by some authors to test the antioxidant activity of these extracts when added to food preparations, and they seem promising enough to inhibit lipid oxidation [61,62,63].

Conventionally, TBA-RS values are used as an index of lipid oxidation in fish and have been correlated with consumer perception of lipid oxidation. This fact is very important from the sensory point of view. Many authors proposed critical TBA-RS values for the association between the degree of lipid oxidation and the detection of rancid odor by panelists or consumers. Djenane *et al*. [37,62] found that a TBA-RS value of 2.00 mg malonaldehyde/kg is closely related to perceptible and unacceptable off-odor of meat.

## 3. Experimental Section

### 3.1. Plant Material and Isolation Procedure

Fresh orange (*Citrus sinensis* L.), lemon (*Citrus limonum* L.) and bergamot (*Citrus aurantium* L.) were collected in the Tizi-Ouzou region (Algeria), in November–December 2012. The whole fresh fruits were then extensively washed with distilled water (20 °C). Only the citrus peels were recovered for subsequent extraction. The citrus peel EOs were obtained from fresh fruit parts by steam hydrodistillation in a Clevenger-type apparatus for 3 h. The yields of extraction were calculated from the initial mass of the plant material in the extractor and the mass of the extract. The EOs obtained were separated from water and preserved in darkness in sealed vials at 1 °C until analysis or use in bioassays.

### 3.2. Essential Oil Analysis

#### 3.2.1. Gas Chromatography

GC analyses of EOs obtained from fresh citrus peels were performed using a Hewlett Packard 6890 gas chromatograph equipped with a flame ionization detector (FID) and a Stabilwax (PEG) polar column (30 m × 0.32 mm i.d.; 1 μm film thickness) (Research Center of Sonatrach, CRD. Boumerdes, Algeria). The operating conditions were as follows: injector and detector temperatures, 250 and 280 °C, respectively; carrier gas, N_2_ at a flow rate of 1 mL/min; oven temperature program, 3 min isothermal at 50 °C, raised at 2 °C/min to 220 °C, and finally held isothermal for 15 min. The identities of the separated components on the polar column were determined by comparing their retention indices relative to aliphatic hydrocarbons injected under the above temperature program with literature values measured on columns with identical polarities.

#### 3.2.2. Gas Chromatography-Mass Spectrometry

The GC-MS analysis was performed using a Hewlett-Packard 6890 series GC system (Agilent Technologies, Madrid, Spain) coupled to a quadrupole mass spectrometer (model HP 5973) equipped with a non-polar HP5 MS capillary column (5% phenyl methylsiloxane, 30 m × 0.20 mm, 0.33 μm film thickness) (CRD, Boumerdes, Algeria). For GC-MS detection, an electron ionization system with ionization energy of 70 eV was used over a scan range of 30–550 atomic mass units (amu). Helium was the carrier gas, at a flow rate of 1 mL/min. Injector and detector MS transfer line temperatures were set at 250 and 280 °C, respectively; the temperature of the ion source was 230 °C. Column temperature was initially kept at 60 °C for 8 min, then gradually increased to 280 °C at 2 °C/min, and finally held isothermal for 30 min. The volume of injections was 0.20 μL of a hexane-oil solution, injected in the splitless mode.

#### 3.2.3. Identification of Components

Retention indices of all the constituents were determined by the Kovats method. Identification of the components was conducted by visual interpretation, comparing their retention indices and mass spectra with data published in the literature [64]. The identity of the components was assigned by matching their spectral data with those detailed in the Wiley 7N, NIST 02, and NIST 98 libraries. The results were also confirmed by the comparison of their retention indices, relative to C7-C29 n-alkanes assayed under GC-MS in the same conditions as the oils. Some structures were further confirmed by available authentic standards analyzed under the same conditions described above. The composition percentage (%) of the oils was computed by the normalization method from the GC peak areas, calculated as the mean value of two injections from each EO.

### 3.3. Antibacterial Activity

#### 3.3.1. Microorganism

The bacterial strain of Gram-positive *S. aureus* was provided by the Spanish Type Culture Collection (STCC). Strain used was *S. aureus* CECT 4459; corresponding to STCC type strain for production of enterotoxin B. Bacterial strain was cultured overnight at 37 °C on Mueller Hinton agar (MHA, Oxoid, Basingstoke, UK). One milliliter (1 mL) of stock culture was standardized through two successive 24 h growth cycles at 37 ± 1 °C in 9 mL of Brain–Heart Infusion Broth (BHIB; Oxoid, Basingstoke, U.K.). After 48 h, 100 μL of the suspension were then inoculated in fresh BHIB and incubated at 37 ± 1 °C for 12 h to obtain a working fresh culture containing about 5 × 10^5^ cfu/mL, determined by measuring transmittance at 600 nm (Spectrophotometer: Spectronic 20 Bausch & Lomb). The strain was kept frozen (−80 °C) in cryovials containing an anti-freezing agent (Cryobanks, Mast, Merseyside-Liverpool, UK) to preserve the viability of the cells during storage and were subcultured every antibacterial test.

#### 3.3.2. Screening Method

Screening of EOs for antibacterial activity was carried out by the agar diffusion method as previously described [36,43], which is normally used as a preliminary check and to select among effective EOs. Petri plates were prepared by pouring 20 mL of Mueller Hinton agar medium and allowed to solidify. Plates were dried for 30 min in a biological safety cabinet with vertical laminar flow and 0.10 mL of standardized inoculums suspension was poured and uniformly spread over the plate. The inocula were allowed to dry for 5 min. To prepare the stock solution of the samples, the pure EOs were dissolved in 5% (*v*/*v*) Dimethyl Sulfoxide (DMSO) (Sigma Aldrich^®^-Química, Madrid, Spain). Then sterile filter paper disks (6 mm diameter, Filter LAB ANOIA, testing paper, Barcelona, Spain) were impregnated with 5 μL EO, using a capillary micro-pipette (Finnpipette^®^, Thermo Fischer Scientific Inc., Vantaa, Finland). The plates were left 15 min at room temperature, to allow the diffusion of the EO, and they were incubated at 37 °C for 24 h. At the end of the period, the diameter of the clear zone around the disc was measured with a caliper (Wiha dialMax^®^ ESD-Uhrmessschieber, Schonach, GmbH) and expressed in millimeters (mm: disk diameter included) as its antimicrobial activity. The sensitivity to the different EOs was classified by the diameter of the inhibition halos. Negative controls were prepared using the same solvent employed to dissolve the samples. Standard reference antibiotic, chloramphenicol (10 μg/disc) was used as positive control in order to test the sensitivity of the tested microorganism. Each assay in this experiment was replicated three times.

#### 3.3.3. Determination of the Minimum Inhibitory Concentration

The MIC values were also studied for the target bacterium which was determined as sensitive to the EOs in disc diffusion assay, as described in above Section. The inoculum of *S. aureus* was prepared from 12 h broth cultures and suspension was adjusted to 0.50 McFarland standard turbidity to give a final density of 5 × 10^5^ cfu/mL. Citrus EOs dissolved in 0.50% DMSO were first diluted to the highest concentration (64 μL/mL) to be tested, and then serial twofold dilutions were made in a concentration range from 64 to 0.02 µL/mL in 10 mL sterile test tubes containing MH broth. MIC values of lemon and bergamot EOs against *S. aureus* were determined based on a microwell dilution method. The 96-well plates (Iwaki brand, Asahi Techno Glass, Tokyo, Japan) were prepared by dispensing into each well 95 μL of MH broth and 5 μL of the inoculum. A 100 μL aliquot from all EOs extracts initially prepared at the concentration of 64 μL/mL was added into the first wells. Then, 100 μL from their serial dilutions was transferred into consecutive wells. The last well containing 195 μL of nutrient broth without compound and 5 μL of the inoculums on each strip was used as negative control. The final volume in each well was 200 μL. Chloramphenicol (Sigma-Aldrich^®^, Madrid, Spain) at the concentration range of 64–0.02 μL/mL was prepared in MH broth and used as standard antibiotic for positive control. Contents of each well were mixed on a plate shaker at 300 rpm for 20 s and then incubated at 37 °C for 48 h. After incubation under agitation, the wells were then examined for evidence of growth and MICs (µL/mL) values were determined as the lowest EO concentration that inhibited visible growth of the tested microorganism, which was indicated by absence of turbidity. The negative control was set up with DMSO in amount corresponding to the highest quantity present in the test solution (0.50%). The tests were performed in triplicate and repeated twice.

### 3.4. Application in Sardine

The sardine retailed and displayed in wooden lockers (prohibited by the government of Algeria, Journal Officiel, 20 June 2010) from a local supermarket (Tizi-Ouzou, Algeria) was freshly purchased and transported to the laboratory under refrigerated conditions within 30 minutes, then headed and gutted, cleaned, rinsed with sterile deionized water, drained, and preserved in refrigerator at 1 °C until use.

#### 3.4.1. Inhibitory Effect of the EOs against *S. aureus* Inoculated in Sardine

Prior to sardine inoculation with *S. aureus* and the addition of EOs, fish muscles were also examined for any contamination by bacteria (aerobic psychrotrophic flora) and the tested pathogen (results not shown). In order to evaluate the antimicrobial activity of EOs in a fish system, a sufficient amount of fresh sardine was prepared following good practices, and was tested using 1 × MIC and 4 × MIC values found for bergamot and lemon EOs. Two individual triplicate of each sample were performed in all cases. For microbiological study, sardine samples were placed in individually stomacher bags and inoculated with strains of *S. aureus* at a level of 3.5 log_10_ cfu/g in the exponential phase (6–12 h). The inoculated samples were homogenized to ensure proper distribution of the pathogen. Following homogenization, the individual EO was added except control to the inoculated samples at 1 × MIC and 4 × MIC values. To attain uniform distribution of the added compounds, treated sardine samples were placed into polystyrene trays further homogenized, as previously described. The sample controls were sprayed with sterile distilled water. All samples from all treatments were wrapped in a pouch made of a polyethylene and polyamide laminate. The samples were stored in the dark at 8 ± 1 °C for 7 days, simulating an actual Algerian refrigeration storage. Microbial analyses of samples for populations of *S. aureus* were carried out at 2 days intervals up to the 7^th^ day of abuse refrigerated storage.

#### 3.4.2. *S. aureus* Enumeration

Microbiological analyses of samples for populations of *S. aureus* were carried out at 2 days intervals up to the 7th day of abuse refrigerated storage (8 ± 1 °C). At each sampling time, samples (25 g) of minced beef in the stomacher bags were aseptically added with 225 mL of 0.10% sterile peptone water. The contents were macerated in the stomacher (Stomacher 400-Circulator. Seward. Worthing, UK) for 1 min at room temperature. Resulting slurries were serially diluted (1:10) in 0.10% sterile peptone water. Sample dilutions (0.10 mL) were spread plated on appropriate media in triplicate. The selective media used for isolation of *S. aureus* was Baird-Parker agar (Oxoid; CM275) supplemented with Egg Yolk-Tellurite emulsion (Oxoid; SR054C). The plates were incubated aerobically 37 °C for 48 h. Counts were expressed as the log_10_ of colony forming units (cfu) per g.

#### 3.4.3. Antioxidant Effect of the EOs in Treated Sardine

To establish a protocol to test the effectiveness of the EOs on sardines, the lowest EOs concentration that applied to the sardine and did not alter the organoleptic characteristics was determined. The choice was made after conducting preliminary sensory tests, during which various samples of 100 g of sardine are added with different concentrations of EOs (1 and 4 × MIC values).

For oxidative study, the remainder fish samples were placed into polystyrene trays. The samples were divided into three groups. The first group (control) was sprayed with sterile distilled water. The final 2 groups were similarly sprayed respectively with 1 and 4 × MIC values (corresponding to 500 and 2000 ppm from lemon; 800 and 3200 ppm from bergamot). Samples from all treatments were wrapped and stored under aerobic conditions at 8 ± 1 °C for 7 days. On days 1, 3, 5 and 7 of storage, two packs containing each sample from each group were opened for lipid oxidation analysis.

Lipid oxidation was measured by the 2-thiobarbituric acid (TBA) method of Djenane *et al*. [65]. Fish samples of 10 g were taken and mixed with 20 mL trichloroacetic acid (10%), using an Ultra-Turrax T25 macevator (Janke & Kunkel, Staufen, Germany). Samples were centrifuged at 2300 g for 30 min at 5 °C; supernatants were filtered through quantitative paper (MN 640 W, Machinery-Nagel GmbH & Co. KG, Düren, Germany). 2 mL of the filtrate were taken and mixed with 2 mL of thiobarbituric acid (20 mM); tube contents were homogenized and incubated at 97 °C for 20 min in boiling water. Absorbance was measured at 532 nm. The concentration of the samples was calculated using a calibration curve. TBA-RS values were expressed as mg malondialdehyde/kg sample.

### 3.5. Statistical Analysis

Variance analyses were used to test the significant difference among the results from the antibacterial assays, sensory and chemical analysis (SPSS 10.0 software package, 1995). Means and standard errors (SE) of the samples were calculated. Three replicates were performed for each treatment. Differences between means were tested through LSD and values of *p* < 0.05 were considered significantly different.

## 4. Conclusions

The present study has allowed us to describe the chemical composition of orange, lemon and bergamot EOs, and to examine and compare their antimicrobial and antioxidant activity. The disk diffusion method might not be an adequate tool for selection among EOs with the best antimicrobial activity. However, the determination of the MIC via the microdilution method revealed a different antimicrobial pattern for the three EOs against the microorganism investigated. The results of the bioassays, together with the chemical profile of the EOs, support the possibility of using lemon and bergamot EOs as potent natural preservatives to contribute in the reduction of experimentally inoculated *S. aureus* in Sardine. It can be stated that bergamot oil is highly effective, therefore the use of citrus peel extract is recommended as a natural antioxidant to suppress development of rancidity in sardines during storage.
